# Illegal harvesting and livestock grazing threaten the endangered orchid *Dactylorhiza hatagirea* (D. Don) Soó in Nepalese Himalaya

**DOI:** 10.1002/ece3.7520

**Published:** 2021-05-01

**Authors:** Deep Jyoti Chapagain, Henrik Meilby, Chitra Bahadur Baniya, Shanta Budha‐Magar, Suresh Kumar Ghimire

**Affiliations:** ^1^ Central Department of Botany Tribhuvan University Kirtipur Nepal; ^2^ Department of Food and Resource Economics (IFRO) University of Copenhagen Copenhagen Denmark; ^3^ School of Science Auckland University of Technology Auckland New Zealand

**Keywords:** anthropogenic disturbances, interview survey, orchid, population density, population monitoring, salep, sustainability

## Abstract

Harvesting of orchids for medicine and salep production is a traditional practice, and increasing market demand is spurring illegal harvest. Ethno‐ecological studies in combination with the effect of anthropogenic disturbance are lacking for orchids. We compared population density and structure, and tuber biomass of *Dactylorhiza hatagirea* (D. Don) Soó for three years in two sites: Manang, where harvesting of medicinal plants was locally regulated (protected), and Darchula, where harvesting was locally unregulated (unprotected). Six populations were studied along an elevation gradient by establishing 144 temporary plots (3 × 3 m^2^) from 3,400 to 4,600 m elevations. Mean density of *D. hatagirea* was significantly higher in the locally protected (1.31 ± 0.17 plants/m^2^) than in the unprotected (0.72 ± 0.06 plants/m^2^) site. The protected site showed stable population density with high reproductive fitness and tuber biomass over the three‐year period. A significant negative effect (*p* < .1) of relative radiation index (RRI) on the density of the adult vegetative stage and a positive effect of herb cover on juvenile and adult vegetative stages were found using mixed zero‐inflated Poisson (mixed ZIP) models. The densities of different life stages were highly sensitive to harvesting and livestock grazing. Significant interactions between site and harvesting and grazing indicated particularly strong negative effects of these disturbances on densities of juvenile and adult reproductive stages in the unprotected site. Semi‐structured interviews were conducted with informants (*n* = 186) in the villages and at the ecological survey sites. Our interview results showed that at the protected site people are aware of the conservation status and maintain sustainable populations, whereas the opposite was the case at the unprotected site where the populations are threatened. Sustainability of *D. hatagirea* populations, therefore, largely depends on controlling illegal and premature harvesting and unregulated livestock grazing, thus indicating the need for permanent monitoring of the species.

## Graphical abstract

*Dactylorhiza hatagirea* (D. Don) Soó is an endangered medicinal orchid occurring in the high Himalayan region. Inadequate conservation awareness among the medicinal and aromatic plant users and illegal harvest are major threats to *D. hatagirea*. Sustainability of *D. hatagirea* populations therefore largely depends on controlling illegal and premature harvesting and unregulated livestock grazing.

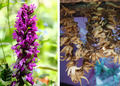

## 1 INTRODUCTION

Globally, 100 to 1,000 species per million become extinct every year, mostly due to anthropogenic habitat deterioration and fragmentation, land use change, urbanization, atmospheric nitrogen deposition, and climate change. Consequently, a large number of species, including many plants, are categorized as vulnerable or threatened (Di Marco et al., 2018; Jacquemyn et al., 2005; Kull & Hutchings, 2006; Pimm et al., 2014). The orchids typify the problem faced by many medicinal and aromatic plant species (MAPs). Orchids, with their complex biology (Rasmussen, 1995; Van der Cingel, 1995), tend to have small and isolated populations and exhibit high sensitivity to environmental changes (Vakhrameeva et al., 2008), and are at greater risk of extinction than most other plant groups (IUCN, 2020; Kull & Hutchings, 2006; Warghat et al., 2013). Human‐mediated disturbances, mainly harvesting and habitat destruction, fragmentation, or loss of habitats are the most significant threats to the survival of orchid populations (Ehrlich, 1988; Laurance & Bierregaard, 1997; Saunder et al., 1991; Zhang et al., 2019). Also, increasing human activities may create novel environments (Zhang et al., 2019) which limit the persistence of orchid populations. Human disturbances may result in the breakdown of ecological connections between orchids and their pollinators and mycorrhiza, changes in edaphic and microclimatic conditions, and introduction of pests and diseases (Fay et al., 2015; Light et al., 2003). Disturbances may interrupt interspecific interactions leading to reduced reproductive output and eventually altering the plant demographic dynamics (Steffan‐Dewenter et al., 2006). Studies have shown that, generally, the magnitude of disturbance impacts on plant populations depends on plant life stage and features of their reproductive system (Calvo, 1990). In the case of orchid populations, the severity of disturbance impacts also depends on the level of specificity of plant–animal interactions (e.g., interactions with pollinators) and the availability of sites suitable for seedling recruitment (Schulze et al., 2019).

The distribution and abundance of orchid populations depend on a suite of biological and ecological factors including seed production and dispersal, recruitment, availability of mycorrhizal fungi, and appropriate environmental conditions (McCormick & Jacquemyn, 2014). However, in case of the smallest orchid populations the seed output may be insufficient to ensure their long‐term persistence (Faast et al., 2011). The life stage dynamics of orchid populations further depends on the elevation of their habitat. Environmental conditions and interactions associated with altitude play a significant role in the composition and distribution of orchid populations (Djordjević & Tsiftsis, 2020; Djordjević et al., 2016, 2020; Jacquemyn et al., 2005). Alpine and subalpine grasslands suffer from reduced nutrient availability and harsh environmental conditions, and plants growing in these habitats presumably develop adaptive coping strategies (Chapagain et al., 2019). Disturbance regimes, such as harvesting, grazing, trampling, and fire, also play influential positive (Chen et al., 2014; Dai et al., 2019) or negative (Chapagain et al., 2019; Kreziou et al., 2015) roles in determining the growth and persistence of plants and could be important elements of an optimal grassland management strategy for alpine meadows. Harvesting of whole plants or plant parts affects reproduction, survival, and growth and thereby also affects plant population dynamics (Gaoue et al., 2013; Ghimire et al., 2005, 2008; Huai et al., 2013; Ticktin, 2004). The extent of harvest impacts on plant populations, however, varies depending on habitat conditions, plant growth strategies, regeneration patterns, and microbial interaction, such as mycorrhizal association (Gaoue et al., 2013; Ticktin, 2015).

Inherently slow growth, high habitat specificity, dependency on pollinators, need of mycorrhiza for reproduction and germination, narrow range of ecological substitution options, unsustainable exploitation, and climate change are major challenges for the growth and development of orchids, such as *Dactylorhiza hatagirea* (Dhiman et al., 2019; Hinsley et al., 2017; Hutchings et al., 2018; Rasmussen & Rasmussen, 2018; Reiter et al., 2017; Shrestha et al., 2021; Yeung, 2017). Due to a marked decline in its natural populations, *D. hatagirea* has been listed as an endangered species in Nepal by Conservation Assessment and Management Plan (Bhattarai et al., 2001). According to the Forest Act of Nepal (2019), collection, use, sale, trade, and export of *D. hatagirea* are prohibited, and the species is strictly protected in list I of Government of Nepal (Go, 2011). It is also listed under appendix II in Convention on International Trade in Endangered Species of Wild Fauna and Flora (CITES, 2020). Nevertheless, due to its high medicinal potency, *D. hatagirea* is still collected illegally at all life stages and traded to, especially, India and China (Olsen & Helles, 1997; Subedi et al., 2013), which has pushed local populations toward extinction (Manandhar, 2002).

Given that many orchids like *D. hatagirea* are currently threatened or endangered, a better understanding of the factors that influence orchid population ecology and dynamics may be critical to their long‐term conservation (Shefferson et al., 2020). Formulation of strategies for conservation of a species requires a sound knowledge of environmental factors, population ecology, and demographic parameters (Margules & Pressey, 2000). Disentangling factors determining successful orchid establishment and its persistence under changing conditions is a major challenge. For the long‐term conservation of endangered orchids, the development of concrete conservation plans based on indigenous knowledge, long‐term monitoring, genetic analysis, and scientific inputs is crucial (Dobriyal et al., 2002; Jacquemyn et al., 2007). Endangered orchid species are in need of specific conservation actions (Charitonidou et al., 2019; Mincheva & Kozuharova, 2018; Tsiftsis et al., 2011), and more attention should be paid to the management of existing sites of orchids (Stipkova & Kindlmann, 2015).

Thus, the objectives of this paper are to (a) analyze the variation in population density, structure, and tuber production in *D. hatagirea* over three years in two sites subjected to different levels of anthropogenic disturbances, (b) study the impact of elevation and anthropogenic disturbances on the population density of *D. hatagirea*, (c) examine the interaction between site and environmental factors (harvesting, grazing, and herb cover) and its effect on population density of *D. hatagirea*, and (d) study the socio‐cultural role of *D. hatagirea* and assess people's perception of its status.

To meet these objectives, we identified a locally unregulated site in the western part of Nepal and a well‐managed, locally regulated site in the central part of Nepal. At these sites, we established permanent plots at different elevations and carried out a survey among local people.

## 2 MATERIALS AND METHODS

### 2.1 Study area

**FIGURE 1:**
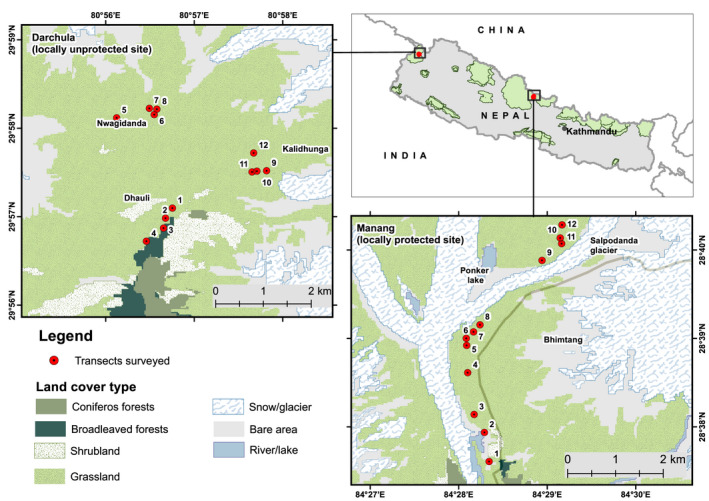
Map of the study area

This study was carried out in two sites: (i) Lolu‐Pilkanda (N29°60.095′ and E080°56.754′ to N29°57.719′ and E080°57.672′) within Api Nampa Conservation Area (ANCA) in Darchula District, northwest Nepal, and (ii) Bhimthang (N28°37.607′ and E084°28.343′ to N28°40.284′ and E084°29.166′), located within the strip of land separating Annapurna Conservation Area (ACA) and Manaslu Conservation Area (MCA) in Manang District, north‐central Nepal (Figure 1).

The Lolu site, which lies in the upper Chamelia valley within ANCA, experiences high human pressure related mainly to livestock grazing and commercial harvesting of medicinal and aromatic plants (MAPs) (DNPWC, 2015). The site is managed by ANCA under the Department of National Parks and Wildlife Conservation (DNPWC) of the Government of Nepal, and due to poor implementation of regulatory mechanisms, the human exploitation of natural resources at this site is very heavy (Pyakurel et al., 2018). Further, the local community has not taken any particular initiatives to conserve the area. Commercial and illegal trade of MAPs from the area has increased drastically in the last few decades (Pyakurel et al., 2018). Hence, the Lolu site is hereafter referred as the locally “unprotected site.”

ANCA is the youngest and the most remote conservation area of Nepal. The climate is temperate to nival with an annual average precipitation of 2,100 mm and annual mean minimum and maximum temperatures of 4.7°C and 27°C, respectively (DNPWC, 2015). Important livelihood activities are collection and trade of high‐value MAPs, notably *Ophiocordyceps sinensis*, *Dactylorhiza hatagirea, Fritillaria cirrhosa*, *Neopicrorhiza scrophulariiflora* (Pouliot et al., 2018; Pyakurel et al., 2018), and traditional mountain farming systems tightly integrated with transhumance and other livestock systems (DNPWC, 2015). The area has legal permission for commercial harvest of MAPs, and over 25,000 collectors from different parts of western Nepal harvest valuable MAPs from rangelands within ANCA (DNPWC, 2015).

The Bhimthang site, which lies in Gyasumdo valley (lower Manang), experiences low human pressure in terms of livestock grazing and commercial and illegal harvesting of MAPs. This site is rich in biodiversity and natural beauty and is located in one of the major tourist areas of Nepal. Thus, tourism is the major source of income for the local community. The area is managed by the community in collaboration with Annapurna Conservation Area Project (ACAP), and the exploitation of valuable MAPs is very limited due to strict regulation by the community (NTNC, 2016). The locals are involved in patrolling to prevent illegal harvesting of MAPs during the maturation period. Further, the local people practice rotational harvest (one or two years depending on the availability of MAPs) of highly valued MAPs (NTNC, 2016). Therefore, the Bhimthang site is hereafter referred to as the locally “protected site.”

At the protected site in Manang, the climate varies from temperate to nival and is influenced by the summer monsoon. The annual mean minimum and maximum temperatures are 4.7°C and 16.8°C, respectively, and the average annual precipitation is 972 mm (DNPWC, 2015). The basic livelihood activities include and combine traditional mountain farming systems, transhumance and animal husbandry, small‐scale trade at lower altitude during winter, tourism, and the collection and trade of highly valued MAPs (Chhetri, 2014; Subedi & Chapagain, 2013).

The study sites differ with respect to edaphic, topographic, and substrate conditions. The unprotected site has a silty‐loamy soil and is rich in herbs and grass and also has some bare ground cover, while the protected site has a sandy‐loamy soil and is rich in moss, lichen, litter, rock, and scree cover. *D. hatagirea* is found on slopes ranging between 1 and 66° at the unprotected site and 4–65° at the protected site. The sites do not vary much in terms of relative radiation index (RRI) (Table S1: Appendix S1). The unprotected site is subjected to higher anthropogenic pressure as revealed by higher disturbance scores (harvesting, grazing, trampling, and animal droppings) than observed at the protected site.

### 2.2 Study species

**FIGURE 2:**
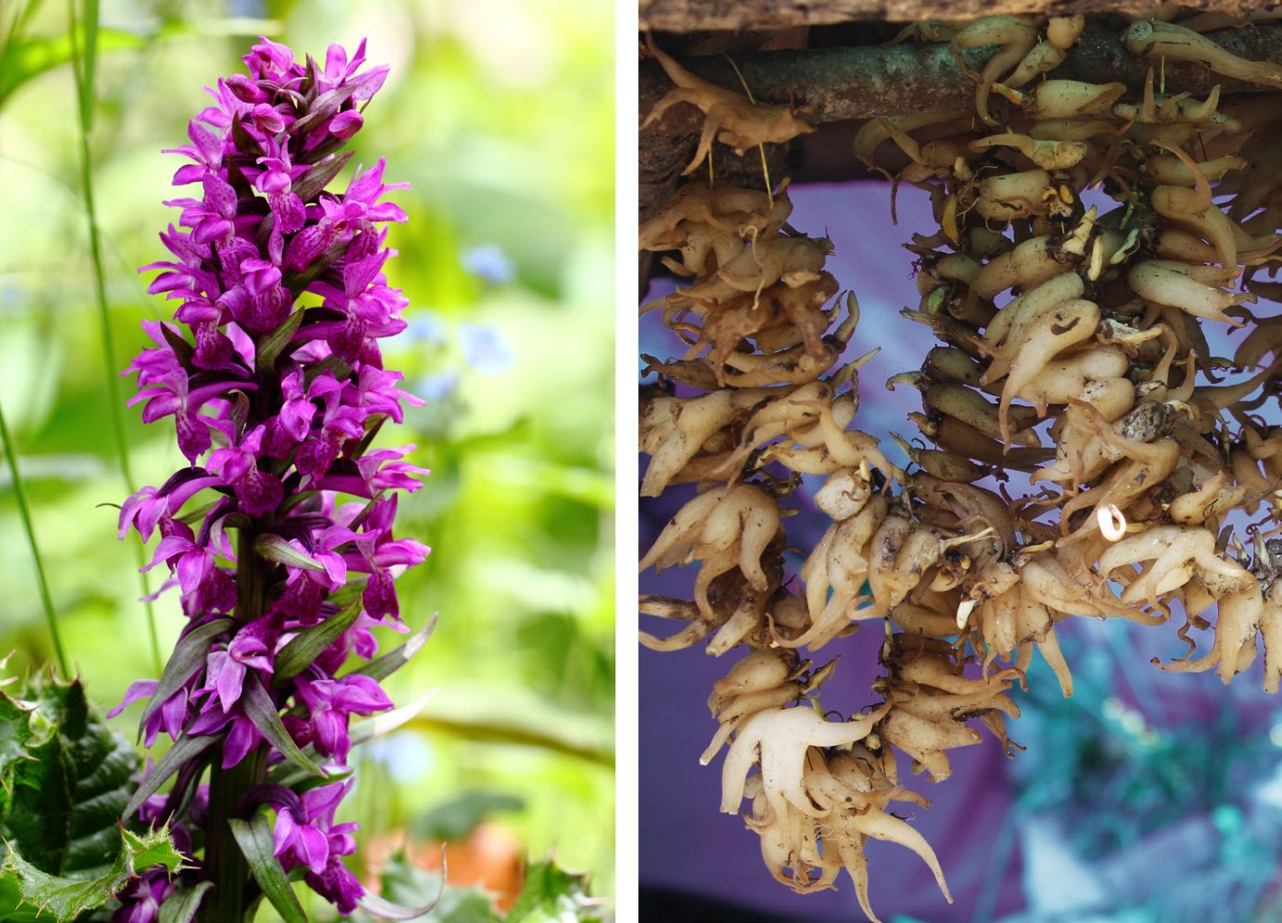
Left: Blooming *Dactylorhiza hatagirea* (D. Don) Soo. Right: Tubers of *Dactylorhiza hatagirea* beaded for drying by the locals (PC: Chandra Kanta Subedi)

*Dactylorhiza hatagirea* (D. Don) Soó is locally known as Panchaunle (“five fingered hand”). It is distributed in Nepal, India, Bhutan, Pakistan, China, Afghanistan, and Mongolia (Roskov et al., 2020). *D. hatagirea* is an erect perennial herb (30–90 cm tall) that grows in moist alpine meadows and forest gaps between 2,800 and 4,600 m asl (Ghimire et al., 1999) favoring high soil Ca content (Thakur et al., 2020). It bears palmate tubers, 5–7 lanceolate or oblong leaves, which are progressively smaller toward the top, and has a robust stem (Figure 2, left). The inflorescence is up to 15 cm long, with a large number of densely packed flowers. Flowers are resupinate and purple to light pink, arranged around the rachis, resembling a hyacinth. Capsules bear thousands of dust‐like seeds. Seeds are minute and have no endosperm and therefore lack storage reserves, and the orchid partly depends on the mycorrhizal fungi *Rhizoctonia* for nutrition (Giri & Tamta, 2012; Kalimuthu et al., 2007; Warghat et al., 2014). The rate of vegetative propagation is very slow and seed germination in nature is very poor, that is, 0.2%–0.3% (Vij, 2001).

The tubers of *D. hatagirea* (Figure 2, right) yield a high quality salep (a beverage made from the powder of the orchid tuber), which is used as an aphrodisiac or a nutritive and restorative tonic, and are also eaten raw as a farinaceous food (Baral & Kurmi, 2006; Sood et al., 2005; Thakur & Dixit, 2007; Vij, 1995; Watanabe et al., 2005). It is also used in the treatment of diabetes, chronic diarrhea, dysentery, coughs, hoarseness of voice, paralysis, fractures, during convalescence and to correct malnutrition (Das, 2004; Singh & Duggal, 2009). The whole plant possesses antibacterial properties and is used in curing various bacterial diseases (Ranpal, 2009). Tubers of *D. hatagirea* contain a wide range of chemical compounds including dactylorhins, dactyloses, glucosides, starch, and albumin (Kizu et al., 1999; Lama et al., 2001). Recent findings indicate that *D. hatagirea* also has anticancerous properties (Popli, 2017).

### 2.3 Sampling design used in the vegetation survey

This research is part of a long‐term study carried out between 2015 and 2017 to understand the population dynamics of *D. hatagirea* in ANCA and Manang (research permit issued by Department of National Park and Wildlife Conservation [DNPWC]).

We sampled the vegetation in two ways: (i) To meet the first objective regarding variation in the populations over time, we established three permanent plots (20 × 20 m^2^) in each population at the two study sites. (ii) Similarly, to meet the second and third objectives regarding impacts of environmental variables on population density, we established a total of 144 temporary plots (3 × 3 m^2^), 72 at each site, from the lowest to the highest elevation to cover the whole distributional range of *D. hatagirea* at the study sites.

In each site, we subjectively identified three fairly dense populations where all life stages were present so that the population could be monitored in the following years (as a part of PhD dissertation of the first author). In each population, we established three randomly located permanent 20 m × 20 m plots and divided each plot into four 10 m × 10 m subplots. In each subplot, all plants were tagged in 2015, categorized with regard to life stage, and monitored in 2016 and 2017.

We established the temporary plots using the methods described by Chapagain et al. (2019). At each site, we identified three populations of *D. hatagirea* along an elevation gradient ranging from 3,400 to 3,900 m along the Upper Chamelia valley of ANCA and 3,400–4,600 m along the Gyasumdo valley in Manang (Figure 1, Table S1: Appendix S1). In each population, we established four transects at a minimum vertical distance of approximately 100 m. In each transect, we established six (3 m × 3 m) plots at a minimum horizontal plot to plot distance of 10 m. Each plot was divided into nine 1 m × 1 m subplots, and the four corner subplots were systematically sampled and measured.

For each plot, the geographical location (latitude and longitude) and topographical characteristics (elevation, slope, and aspect) were recorded and used to calculate the relative radiation index (RRI) (Oke, 1987; Vetaas, 1992a, 1992b). In each subplot, the ground cover (%) for vascular plants (grasses, herbs, and shrubs), nonvascular plants (lichens and bryophytes), litter, bare ground, rock, and scree cover were estimated using standard methods (Pauli et al., 2015). Disturbance (harvesting, trampling, grazing, and animal droppings) scores ranging from 0 (none) to 4 (very high) were recorded for each subplot after careful observation of the evidence, for example, large holes caused by excavation, wilted or fresh uprooted aerial parts, tuber fragments, browsed plant parts, defoliated aerial parts, and animal droppings.

We classified the individual plants into four life stage classes based on the number and size of leaves and presence of reproductive structures. The four stages are as follows: seedlings (Sd; leaf breadth ≤1 cm, leaf number = 1–2), juveniles (Jv; leaf breadth ≥1 cm, ≤2 cm, leaf number = 2–3), vegetative adults (Adv; leaf breadth ≥2 cm, leaf number >2, nonflowering), and reproductive adults (Adr; flowering or fruiting individuals). Individuals at different stages were counted in each subplot to calculate the density. The population structure was described as the proportion of each life stage within the studied population.

To estimate reproductive traits, we selected fifteen mature individuals from each population at the two sites and recorded the reproductive traits (number of flowers and fruits). For the estimation of dry biomass of tubers, we recorded the weight of fifteen dried tubers of *D.hatagirea* from the local MAPs collectors.

### 2.4 Interview survey

Semi‐structured interviews were conducted during 2015–2017 among 117 persons in the unprotected site (Darchula) and 69 persons in the protected site (Manang). The informants were local MAPs users, leaders, teachers, and students in the villages, and hotel owners, local tourist guides, and cattle herders working in the sites where we carried out our ecological survey. We explained the goal of our research and obtained the informants’ consent before starting the interview. The informants were also informed about their right to withdraw their consent at any stage of the interview. Informants’ responses were documented by written notes and in possible cases were also supplemented by voice recording, with the permission of the informants.

We asked the informants to comment on the abundance of *D. hatagirea*, collectors (villagers or outsiders), reasons for collecting the plant (local use or commercial purpose), local uses, the collection (areas and time of collection), when they started collecting, indigenous knowledge transfer practices, special tools, prices, qualities, markets (to whom they sold), processing after collection, and their other sources of income. We also asked questions concerning nature conservation; changes observed in the biotope, perceived trends in populations of the species, causes of population change, threats, conservation status, protection measurements, and practices that could ensure survival and sustainable management.

### 2.5 Data analysis

A relative radiation index (RRI), which is a relative measure of the exposure to solar radiation at noon at a specific location (Oke, 1987; Vetaas, 1992a, 1992b), was calculated for each plot as a function of aspect, latitude, and slope:

$$\text{RRI}=\cos\left(180^{\circ }‐\Omega \right)\times \sin\left(\beta \right)\times \sin\left(\Phi \right)+\cos\left(\beta \right)\times \cos\left(\Phi \right),$$

where Ω is aspect (slope azimuth in degrees), Φ is latitude (degrees), and *β* is slope inclination (degrees).

The densities of different stages (seedling, juvenile, vegetative, and reproductive adults) were compared by Kruskal–Wallis tests, and the reproductive traits were compared among the three populations in each of the two sites using one‐way ANOVA.

Direct field observations confirmed the plant as rare in the study site, and we therefore expected that the data collected would exhibit a large number of zeros. We tried with different model alternatives but based on the Akaike information criterion (AIC) the best fit was obtained using mixed zero‐inflated Poisson (ZIP) models. The mixed ZIP model allowed us to analyze relationships between density of *D. hatagirea* plants at different stages and a set of independent variables including population, cover of shrubs or herbs, relative radiation index (RRI), and anthropogenic disturbance indicators such as harvesting, trampling, grazing, and animal droppings.

We prepared ten sets of candidate models using the glmmTMB package (See Appendix S2) and finally prepared an average model based on the set of five best candidate models (selected on the basis of delta AIC) using the MuMIn package (Barton, 2018). More specifically, the final average models were prepared using five models with delta AIC values ≤402 for the seedling stage, ≤748 for the juvenile stage, ≤645 for the adult vegetative stage, and ≤763 for the adult reproductive stage. The full models were in all cases expressed as:

$\text{Density}\;\left(\text{of a particular stage of}\;D.\;hatagirea\right)=a+b\;\left(\text{population}\right)+c_{1}{\text{RRI}}_{\mathrm{ij}}+c_{2}{\text{Herb cover}}_{\mathrm{ij}}+c_{3}{\text{Harvesting}}_{\mathrm{ij}}+c_{4}{\text{Trampling}}_{\mathrm{ij}}+c_{5}{\text{Grazing}}_{\mathrm{ij}}+c_{6}{\text{Animal dropping}}_{\mathrm{ij}}$where *a* (intercept), *b* (population), and *c*_1_…*c*_6_ are fixed model parameters. *i* = 1…144 is the plot (included as a random effect); *j* = 1…4 is the subplot. The population variable had six categories, three at each site (see the section Study area). Confounded variables were excluded from the analysis. All the tests were conducted using the R version 3.5.3 (R Development Core team, 2017).

## 3 RESULTS

### 3.1 Variation in population density and structure

The population density of *D. hatagirea* at the unprotected site ranged from 0.60 to 0.79 individuals/m^2^ while the density ranged from 0.70 to 2.16 individuals/m^2^ at the protected site. Population densities were highest in mid‐elevation populations at both sites (Table 1). At the unprotected site, all populations showed highest densities for the juvenile and adult vegetative stages, whereas at the protected site, the density of the adult reproductive stage was mostly higher than for the juvenile and adult vegetative stages (Table 1). At the protected site, the variation of density among the populations was significant (Kruskal–Wallis test, *p* < .05), overall, and for all stages except the adult vegetative stage, but at the unprotected site, there was no significant variation among populations (*p* >.05).

**TABLE 1:** Population density (m^−2^) for different life stages of *Dactylorhiza hatagirea* in populations in the locally unprotected (Darchula) and locally protected (Manang) sites

Population	Elevation (m asl.)	Life stage class^a^				Total
		Sd	Jv	Adv	Adr	
Dhauli^up^	3,605	0.05 ± 0.03	0.20 ± 0.03	0.20 ± 0.04	0.13 ± 0.02	0.60 ± 0.06
Nwagidanda^up^	3,799	0.06 ± 0.02	0.39 ± 0.09	0.21 ± 0.04	0.12 ± 0.03	0.79 ± 0.10
Kalidhunga^up^	3,976	0.08 ± 0.03	0.25 ± 0.04	0.28 ± 0.07	0.17 ± 0.03	0.78 ± 0.12
Mean^up^		0.07 ± 0.01	0.29 ± 0.03	0.23 ± 0.03	0.14 ± 0.01	0.72 ± 0.06
*χ^2^* Value		0.70	2.56	0.98	1.39	2.57
*p*‐Value		.70	.28	.61	.50	.28
Bhimthang^p^	3,713	0.17 ± 0.05	0.46 ± 0.10	0.18 ± 0.07	0.27 ± 0.06	1.07 ± 0.17
Ponker Hill^p^	4,046	0.44 ± 0.17	0.49 ± 0.14	0.40 ± 0.10	0.82 ± 0.17	2.16 ± 0.40
Salpodanda^p^	4,437	0.03 ± 0.02	0.09 ± 0.03	0.19 ± 0.05	0.40 ± 0.04	0.70 ± 0.07
Mean^p^		0.21 ± 0.07	0.34 ± 0.07	0.26 ± 0.04	0.50 ± 0.07	1.13 ± 0.17
*χ^2^* Value		13.62	8.86	3.09	10.63	14.24
*p*‐Value		<.01	<.05	.21	<.01	<.01
Combined						
*χ* ^2^ value		18.64	14.82	3.92	42.99	21.77
*p*‐Value		<.001	<.01	.41	<.0001	<.001

Note

Densities are stated as mean ± *SE*. *χ* ^2^ and *p*‐values were based on Kruskal–Wallis test, *df* = 5, *n* = 144.

up = locally unprotected site, p = locally protected site.

^a^ Life stage classes—Sd: seedling; Jv: juvenile; Adv: adult vegetative; Adr: adult reproductive.

**FIGURE 3:**
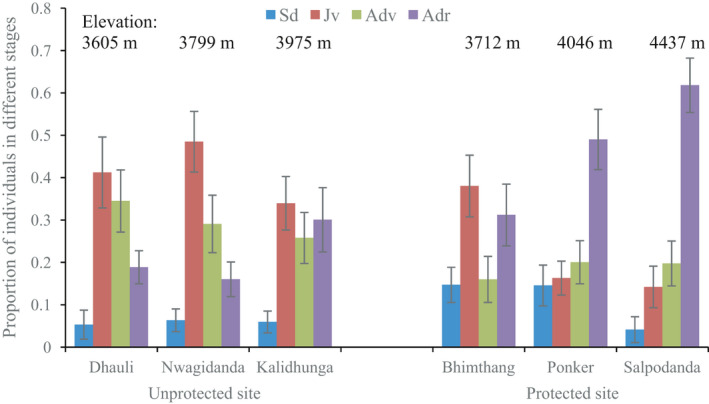
Population structure of *Dactylorhiza hatagirea* in (left) locally unprotected and (right) locally protected sites. Life stage classes: Sd = Seedling, Jv = juvenile, Adv = adult vegetative, and Adr = adult reproductive

**FIGURE 4:**
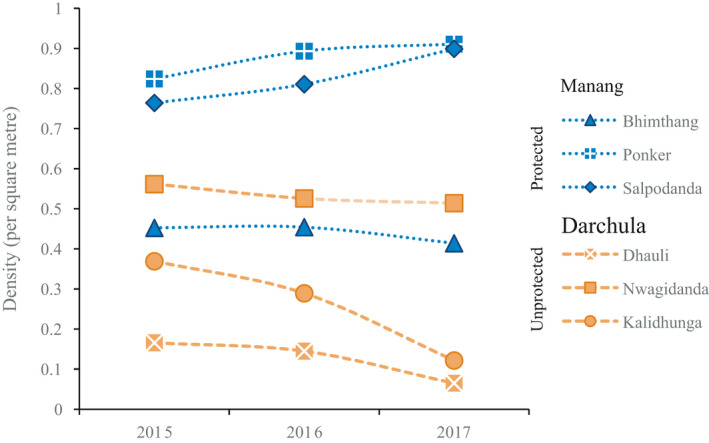
Density of *Dactylorhiza hatagirea* at the protected (Manang) and unprotected (Darchula) sites in 2015, 2016 and 2017

The population structure varied between the sites (Figure 3). For all populations, the proportion of seedlings was lower at the unprotected site than at the protected site. The proportion of juvenile and adult vegetative plants was the highest at the unprotected site, while the proportion of the adult reproductive stage was highest at the protected site. Comparing mean densities across three consecutive years (2015–2017), a drastic decrease was observed at the unprotected site (approximately by one third), while almost no change was seen at the protected site (Figure 4).

### 3.2 Variation in reproductive traits and tuber production

**FIGURE 5:**
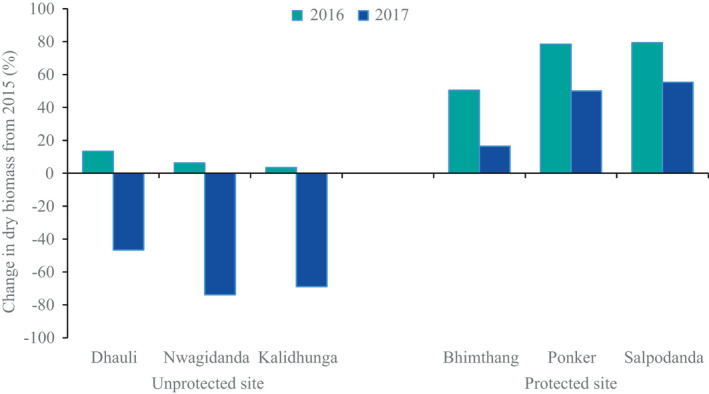
Variation in tuber production of *D. hatagirea* during 2015 to 2017 in populations in the locally unprotected (first three in the graph) and locally protected sites (last three in the graph)

At the protected site, the reproductive output and tuber biomass were about three times higher than at the unprotected site. In comparisons between populations, the reproductive traits (number of flowers, number of fruits, and total reproductive output per individual) were found to decrease from the lowest to the highest elevation at both sites (Table 2). The dry biomass of daughter tubers showed similar trends at both study sites. A comparison of tuber production across three consecutive years (2015, 2016, and 2017) showed reduced tuber production in 2017 at the unprotected site and increasing tuber production at the protected site (Figure 5).

**TABLE 2:** Variation in reproductive output of *Dactylorhiza hatagirea* in populations in the locally unprotected (Darchula) and locally protected (Manang) sites

Population	Number of flower per individual	Number of fruit per individual	Total reproductive output per individual	Dry weight of daughter tuber (g)
Dhauli^up^	15.27 ± 3.06	27.87 ± 1.94	39.07 ± 4.30	0.73 ± 0.08
Nwagidanda^up^	14.89 ± 1.20	26.33 ± 1.92	34.93 ± 2.67	0.63 ± 0.13
Kalidhunga^up^	13.11 ± 3.65	24.5 ± 2.07	27.40 ± 2.96	0.61 ± 0.09
Total				
*F*‐value	0.49	2.22	9.47	3.06
*p*‐Value	.49	.14	.15	.09
Bhimthang^p^	29.5 ± 2.39	40.7 ± 3.05	71.2 ± 2.93	1.74 ± 0.13
Ponker Hill^p^	27.9 ± 3.27	36.9 ± 2.18	64.8 ± 4.40	1.42 ± 0.16
Salpodanda^p^	21.9 ± 1.61	31.4 ± 1.84	53.3 ± 2.32	1.17 ± 0.14
Total				
*F*‐value	9.78	5.11	12.89	8.82
*p*‐Value	.01	.02	.00	.00
Combined				
*F*‐value	0.26	29.8	29.8	92.3
*p*‐Value	.61	.00	.00	.00

Note

Values are stated as mean ± *SE*. *F* and *p*‐values were based on one‐way ANOVA, *df* = 5, *n* = 144.

up = locally unprotected site, p = locally protected site.

### 3.3 Effect of environmental variables on densities of different life stages and their interaction among the sites

Mixed zero‐inflated Poisson (mixed ZIP) models showed significant negative effects of harvesting on the densities of juvenile and adult stages (both vegetative and reproductive). Grazing had a significant negative effect on juvenile (*p* < .001) and adult vegetative (*p* < .05) stages. Herb cover showed significant positive effects (*p* < .05) on the juvenile and adult vegetative stages. The relative radiation index had a weakly significant negative effect (*p* < .1) on the density of the adult vegetative stage. The zero‐inflation model component was significant only for the adult reproductive stage (Table 3).

**TABLE 3:** Mixed zero‐inflated Poisson regression models for the density (m^−2^) of seedling, juvenile, adult vegetative, and adult reproductive stages of *Dactylorhiza hatagirea*

Life stages	Average model	ZI component	Locally protected site (Manang)			Locally unprotected site (ANCA)							
			Count component										
		Zi	Bhimthang: Intercept	Ponker Hill	Salpodanda	Dhauli	Nwagidanda	Kalidhunga	Herb cover	RRI	Harvesting	Grazing	Animal droppings
Seedling	Full	−0.702 (0.691)	−1.982*** (0.619)	0.537 (0.553)	−2.037** (0.818)	−0.677 (0.876)	−0.740 (0.720)	−0.514 (0.750)	0.009 (0.010)		−0.606 (0.369)	−0.424 (0.394)	0.019 (0.116)
	Conditional	−0.702 (0.691)	−1.982*** (0.691)	0.537 (0.553)	−2.037** (0.818)	−0.677 (0.876)	−0.740 (0.720)	−0.514 (0.750)	0.013 (0.009)		−0.606 (0.369)	−0.606 (0.336)	0.147 (0.292)
Juvenile	Full	−2.150 (1.401)	−0.919** (0.363)	−0.133 (0.346)	−1.854**** (0.470)	−0.070 (0.488)	0.002 (0.381)	−0.213 (0.424)	0.010 (0.007)		−0.367** (0.177)	−0.644**** (0.188)	−0.080 (0.149)
	Conditional	−2.150 (1.401)	−0.919** (0.363)	−0.133 (0.346)	−1.854**** (0.470)	−0.070 (0.488)	0.002 (0.381)	−0.213 (0.424)	0.011** (0.004)		−0.376** (0.169)	−0.644**** (0.188)	−0.197 (0.179)
Adult vegetative	Full	0.122 (0.307)	−1.080* (0.560)	0.953** (0.421)	−0.201 (0.461)	1.026* (0.590)	0.437 (0.456)	0.720 (0.490)	0.009 (0.007)	−0.106 (0.459)	−0.657*** (0.230)	−0.530* (0.290)	−0.014 (0.010)
	Conditional	0.122 (0.307)	−1.080* (0.560)	0.953** (0.421)	−0.201 (0.461)	1.026* (0.590)	0.437 (0.456)	0.720 (0.490)	0.010** (0.006)	−0.594* (0.903)	−0.657*** (0.230)	−0.595** (0.238)	−0.071 (0.217)
Adult reproductive	Full	−0.781** (0.370)	0.894** (0.426)	1.138**** (0.287)	0.394 (0.307)	0.353 (0.413)	−0.106 (0.389)	0.224 (0.373)	0.000 (0.001)	−0.098 (0.411)	−0.801**** (0.202)	−0.057 (0.134)	−0.010 (0.061)
	Conditional	−0.781** (0.370)	−0.894** (0.426)	1.138**** (0.287)	0.394 (0.307)	0.353 (0.413)	−0.106 (0.389)	0.224 (0.373)	0.000 (0.004)	−0.583 (0.853)	−0.801**** (0.202)	−0.177 (0.188)	−0.103 (0.173)

Note

Density of different stages was modeled as a function of herb cover (%), relative radiation index (RRI), and disturbance (harvesting, grazing, and animal droppings), which were assessed using an ordinal 0–4 integer scale. Zi is the intercept in the zero‐inflated component: all other parameters refer to the count component of the model. Parameter estimates with standard errors in brackets for full average and conditional average models. Significance levels are stated as: *p < .1, **p < .05, ***p < .01, ****p < .001.

The interaction effects of harvesting and grazing within population among the sites indicate that the unprotected populations showed significant negative effects of harvesting and grazing, while these effects were not distinct for the protected populations. The herb cover did not show any significant effects within populations among the sites (Table 4).

**TABLE 4:** Mixed zero‐inflated Poisson regression models expressing the interaction effect between site and environmental factors (harvesting, grazing, and herb cover) on density (m^−2^) of seedlings, juvenile, adult vegetative, and adult reproductive stages of *D. hatagirea*

Life stages	Independent variables	Harvesting				Grazing				Herb Cover			
	Model	Intercept: Locally unprotected site	Locally protected site	Harvest	Interaction locally protected * harvest	Intercept: locally unprotected	Locally protected	Grazing	Interaction locally protected * grazing	Intercept: locally unprotected	Locally protected	Herb cover	Interaction locally protected * Herb cover
Seedling	Conditional	−2.561**** (0.540)	0.180 (0.461)	−0.860*** (0.307)	1.012 (1.207)	−2.338**** (0.553)	0.178 (0.488)	−1.011*** (0.348)	0.317 (0.558)	−3.569*** (0.899)	1.209 (0.916)	0.002 (0.011)	−0.004 (0.014)
	Zero Inflation	0.774 (0.549)				−0.789 (0.557)				−0.667 (0.719)			
Juvenile	Conditional	−0.546*** (0.201)	−0.708*** (0.244)	−0.833**** (0.138)	2.250** (0.922)	−0.354* (0.212)	−0.833*** (0.262)	−1.081**** (0.168)	0.757** (0.329)	−1.230*** (0.417)	0.132 (0.472)	0.000 (0.006)	0.000 (0.009)
	Zero Inflation	−1.327**** (0.466)				−1.614**** (0.466)				−0.668 (0.377)			
Adult vegetative	Conditional	−0.200 (0.251)	−0.654** (0.284)	−0.963**** (0.180)	0.903 (0.842)	0.019 (0.263)	−0.787** (0.321)	−1.126**** (0.207)	0.650 (0.408)	−0.667 (0.476)	0.034 (0.541)	0.001 (0.007)	0.006 (0.010)
	Zero inflation	0.093 (0.308)				0.047 (0.308)				0.732*** (0.267)			
Adult reproductive	Conditional	−0.851**** (0.234)	0.378* (0.230)	−0.949**** (0.204)	1.039** (0.501)	−0.660*** (0.256)	0.299 (0.256)	−0.956**** (0.200)	0.732** (0.294)	−1.551**** (0.464)	1.279*** (0.478)	−0.000 (0.007)	−0.002 (0.008)
	Zero inflation	−0.760* (0.389)				−0.666* (0.368)				−0.474 (0.337)			

Note

Parameter estimates with standard errors in brackets. Significance levels are stated as: *p < .1, **p < .05, ***p < .01, ****p < .001.

### 3.4 Interview survey

About forty‐three percent of the MAP users interviewed at the unprotected site (*n* = 117) were aware that *D. hatagirea* is strictly protected, seventy‐one percent were aware of its use value, five percent were aware of its population ecology, and forty‐two percent harvested *D. hatagirea* for local uses to treat cuts and wounds, boils, fractures, and to use it as a tonic. Ninety‐two percent of the informants claimed that illegal harvesting of *D. hatagirea* was a common practice and that the population had decreased drastically over the last few decades. They further disclosed that there are illegal traders in the district headquarters of Darchula who motivate MAP users and cattle herders to harvest *D. hatagirea* by promising to buy the dried tubers. Further, perceived difficulties to collect *Ophicordyceps sinensis* and *Fritillaria cirrhosa* increased the temptation to carry out illegal and premature harvest of *D. hatagirea*. The informants mentioned that for every 1 kg of dried tubers sold, approximately 500–1,000 mature plants are harvested. Eighty‐one percent of the informants are of the view that the major source of their livelihood is MAP collection.

At the protected site, about ninety‐three percent of the MAP users interviewed (*n* = 69) were aware of the harvesting ban, ninety‐eight percent were aware of the species’ use value, nineteen percent were aware of its population ecology, and twenty‐one percent occasionally harvested a few (2–5) individuals of *D. hatagirea* for home use to treat cuts and wounds, burns, and boils and for religious purposes by Buddhist Lamas. About twenty‐four percent were of the opinion that the population is decreasing, while seventy‐three percent thought that the population has been almost constant in the last few decades as the local community is involved in patrolling the area to control illegal collection during the maturation period. Our interview results also revealed that none of the families totally rely on MAP collection for their livelihood as they had access to income from the flourishing tourism. We also observed that awareness programs were run at community level, emphasizing the sustainable use of available MAPs. Further, they also used a specific route to protect sensitive plants from damage caused by grazing and trampling.

## 4 DISCUSSION

The study is the first of its kind in this region, but similar research has been done in Greece (Charitonidou et al., 2019). Our work provides results based on a long‐term study from the Nepal Himalayas and should therefore be of significance to the conservation management of the endangered orchid *D. hatagirea*. Environmental variables and human‐mediated disturbances such as harvesting and livestock grazing had significant effects on the population structure, density, reproductive traits, and tuber production of *D. hatagirea*.

### 4.1 Variation in population density and structure

We recorded a maximum population‐level mean density of 1.31 plants/m^2^ at the protected and 0.72 plants/m^2^ at the unprotected site. The population density at the protected site was thus roughly twice as large as at the unprotected site. Particularly low proportions of seedlings and adult reproductive plants observed at the unprotected site could be due to overharvesting and grazing, since harvesters presumably tend to target the adult reproductive stage and recently established seedlings are sensitive to grazing. Moreover, seedlings browsed by livestock may be hard to find and could therefore be overlooked more frequently than the larger plants characterizing later life stages.

At both sites, we recorded a low proportion of seedlings. This could partly be explained as a consequence of orchids being habitat specific and seedling establishment depending on a suite of environmental factors, which are rarely present at the same time and place (Shefferson et al., 2020). Our results compare well with previous research as small populations have substantially lower viability compared to larger populations, and seedling recruitment rates can be considerably lower in small populations, which results in significantly lower population growth rate and density (Hens et al., 2017; Pellegrino & Bellusci, 2014). The low proportion of reproductive plants observed at the unprotected site compares well with observations made by Pellegrino and Bellusci (2014) for the orchid species *Serapias cordigera*, where human disturbances were noted to have a negative effect on the population size. The traditional practices of transhumance, harvesting, and habitat fragmentation are likely the major anthropogenic factors responsible for reducing the flowering density as well as the population size of *D. hatagirea* in the unprotected site. Human‐induced disturbances like harvesting, grazing, and fire have negative effects on population density and performances (Aguilar et al., 2006; Chapagain et al., 2019; McKinney, 2002); however, there are also examples of the opposite, and Chen et al. (2014) actually reported a positive effect of human disturbance on some orchid species. Our results further suggest that at both sites the density of *D. hatagirea* reached a peak in mid‐elevation populations (3,799 m asl in the unprotected and 4,046 masl in the protected site). This might indicate that we have actually managed to cover the elevation range and that the best habitats are thus found approximately in the middle of the range. It is also consistent with the idea that when examining small populations across environmental gradients, the peak density usually occurs at intermediate levels, as also noted by Chen et al. (2014).

### 4.2 Variation in the reproductive traits

We found reduced reproductive fitness in *D. hatagirea* at the unprotected site. Alterations due to anthropogenic disturbances in natural habitats often reduce the size and density of populations (Aguilar et al., 2006; McKinney, 2002). Anthropogenic disturbances increase the spatial distance between the plant populations as well as between individuals within a population, thereby disrupting insect movement between plants (Öckinger et al., 2009), decreasing pollinator abundance (Liu & Koptur, 2003), and altering their behavior and the frequency of flower visits (Aguilar et al., 2006). This process ultimately decreases the reproductive fitness of the plants (Peterson et al., 2008). The level of inbreeding may be higher in small, isolated populations (Miao et al., 2014) because of the higher rate of selfing and more frequent mating between close relatives. The resulting inbreeding depression can reduce the fitness of the plants compared with those in larger populations. Consequently, decreased outcrossing in small, sparse populations may reduce population fitness, potentially increasing the probability of extinction (Gargano et al., 2009; Stachurska‐Swakoń et al., 2011). The weaker performance in terms of reproductive output at the unprotected site could further be attributed to the interruption of plant development caused by breakage of inflorescences during heavy grazing and trampling. A long history of grazing and trampling at the unprotected site (DNPWC, 2015) might have resulted in the production of reduced or defective reproductive parts. Grazing is likely to affect *D. hatagirea* in numerous ways, both directly by damaging aboveground parts and indirectly by changing habitat characteristics (light intensity, litter accumulation, temperature, etc.) and interaction with other individuals or species (intra‐ and interspecific competition, pollination/herbivory).

The unprotected populations had a lower population size and a lower fruit set than did protected populations, suggesting that the latter populations are better buffered. This could be a consequence of inadequate pollinator visitation in small populations, resulting in insufficient pollen transfer, poor pollination, and lower seed set (Smithson, 2006; Tremblay et al., 2005; Xia et al., 2013). By contrast, larger populations of plants are likely to be more attractive to pollinators, resulting in higher visitation rates and therefore higher pollination success (Mustajärvi et al., 2001). Habitat fragmentation and disturbance lead to the interruption of interspecific interactions, indirectly causing changes in plant demographic dynamics via reduced reproductive output (Shefferson et al., 2020; Steffan‐Dewenter et al., 2006). Some studies have shown that plants in disturbed habitats undergo a reduction in pollination efficiency and reproductive success as well as recruitment and survival rates, all of which negatively affect plant demographic dynamics (Aguilar et al., 2006; Bruna et al., 2009).

Transhumance and MAPs collection are common practices in the alpine and subalpine pastures of the unprotected site and have existed for a very long time (DNPWC, 2015). The site is legally open for commercial harvest of highly valued MAPs, such as *Ophiocordyceps sinensis* and *Fritillaria cirrhosa*, and the locals are highly dependent on the collection and trade of MAPs for their livelihood. A large number of collectors (locals as well as from other parts of the country) enters the site to collect *O. sinensis* and *F. cirrhosa* during the late spring when *D. hatagirea* also emerges. The resulting trampling presumably has a huge negative impact on its growth and development by breaking the aerial parts before fruit maturation and seed dispersal. Besides, collectors who are unable to collect sufficient amounts of *O. sinensis* and *F. cirrhossa* are tempted to illegally harvest the tubers of *D. hatagirea* irrespective of its degree of maturity. Such practices are also common in other parts of the world (Ghorbani et al., 2014; Kreziou et al., 2015). Further, the harvesting of the orchids involves destructive uprooting of the daughter tubers, which kills the plants.

When ascending from lowland to alpine environments in the Himalayas, plant species experience a large variation in abiotic conditions over an extremely short distance (Korner, 2003). With increasing elevation, changes in pressure, temperature, wind speed, UV exposure, and soil properties have been shown to affect different phenological and morphological properties of plants (Djordjević & Tsiftsis, 2020; Hodkinson, 2005), thus also influencing growth and reproductive performance. The decreasing number of reproductive parts observed along the altitudinal gradient could be further attributed to the time of flowering, which is influenced by the ambient temperature and the timing of the snow melt (Kudo & Hirao, 2006).

### 4.3 Effects of different environmental variables on the density of different stages

The occurrence and distribution of orchid species are influenced by environmental and topographical factors such as latitude, altitude, slope, and aspect (Bulafu et al., 2007; Djordjević et al., 2016, 2020). In this study, we observed a very weak negative effect of the Relative Radiation Index (RRI) on the adult vegetative density (*p* < .1) of *D. hatagirea*. Although the effect is weak, it may indicate that *D. hatagirea* prefers growing in humid places. Mixed ZIP models revealed a significant positive effect of herb cover on the juvenile and adult vegetative density. It may be possible to explain this as a consequence of other herbs providing shade and shelter for the growth and development of *D. hatagirea* individuals at different life stages. The harvesting showed significant negative effects on the density of all stages except seedlings, as also observed in other parts of the world (Ghorbani et al., 2014; Kreziou et al., 2015), whereas grazing showed significant negative effects on juvenile and adult vegetative stages only. Contrasting results from other parts of the world also exist. For example, Charitonidou et al. (2019) found that the current level of collection of *Dactylorhiza sambucina* in Greece is not significantly affecting the abundance of this orchid, and Mincheva and Kozuharova (2018) reported that wild orchids are not threatened by harvesting in Bulgaria.

Grazing and trampling adversely affect aboveground parts and disturb the life cycle. In some cases, the underground parts are also exposed and eventually destroyed. The weak positive effect of harvest and grazing on plant density at the protected site for all the different stages could be linked to the narrow range of harvest and grazing intensities observed. By contrast, at the unprotected site, the negative impact of harvesting and grazing on density of different stages was highly pronounced. The prevailing disturbance practices (grazing, trampling, and overexploitation) and lack of awareness of the population ecology and the conservation status of the plant are the major challenges for sustainable management at the unprotected site (Pouliot et al., 2018). This is also in agreement with Poudeyal et al. (2019) who observed that intense human disturbances, especially harvest, played a crucial role as determinants of the density and structure of *Neopicrorhiza scrophulariiflora* populations.

### 4.4 Interview survey

We observed a higher proportion of people at the protected site who knew about the harvest ban and the population ecology and use value of the species than at the unprotected site. The strict enforcement of MAPs harvesting rules by the community at the protected site is the key factor for the maintenance of sustainable populations of *D. hatagirea*. People at the protected site do not allow people from other districts to collect any types of MAPs in their territory (NTNC, 2016) and this helps to maintain a low harvesting pressure in populations of MAPs including *D. hatagirea*. Moreover, the Annapurna Conservation Area Project (ACAP), which has worked in this area for three decades, has also contributed to increase conservation awareness and promote sustainable use of natural resources (Baral & Heinen, 2007).

In contrast, in the unprotected site there is no strict local protection system and people from outside ANCA are also allowed to harvest MAPs. The local MAP users were also found with to have a low level of conservation awareness (in relation to *D. hatagirea*) as also reported for northwestern Greece by Kreziou et al., (2015). Moreover, due to the lack of protection measures and awareness, the MAP users were found to engage in intensive harvesting of *D. hatagirea*, and selling it to local traders (Pyakurel et al., 2018) despite the local legal acts of protection of *D. hatagirea*.

Additionally, a group of locals at the unprotected site has the view that the uncontrolled influx of unaware collectors from different parts of the country exacerbates the exploitation of the alpine vegetation. The prevailing unhealthy competition among MAP collectors in the unprotected site sometimes terminates in social conflicts which promotes the illegal harvest of *D. hatagirea* challenging its persistence. Thus, unregulated harvesting of *D. hatagirea* could be one of the major reasons for decline in the populations of the species.

## 5 CONCLUSION AND CONSERVATION IMPLICATIONS FOR *D. HATAGIREA*

Harvesting and grazing and a low level of awareness about the population ecology and conservation among local people are the major challenges for sustainable development of *D.hatagirea* populations at the unprotected site. Harvesting and grazing showed significant negative effects on the density of different life stages of *D. hatagirea* and played a crucial role in deteriorating plant populations through reduction in reproductive outputs. Hence, this study indicated significantly reduced fruit production and lower productivity in terms of tuber biomass for plants at the unprotected site. Moreover, disturbances have the potential to cause a reduction in number of recruits and adult individuals (both vegetative and reproductive) as intensive destructive harvesting techniques are used at the unprotected site, irrespective of life stage and maturity. Coupled with other prevalent disturbances, the intensive and destructive harvesting may lead to local extinction of the species. Therefore, a good strategy for long‐term conservation of the species would involve strengthening people's knowledge about the population ecology of *D. hatagirea*, increasing the enforcement of current regulations and introducing permanent monitoring of the populations. This study also recommends that governmental and nongovernmental organizations working in the field of conservation help in identifying alternative sources of income for the locals, so that their dependency on MAPs harvesting can be reduced, thus also reducing the pressure on populations of *D*. *hatagirea* and preventing local extinction of the species.

## CONFLICT OF INTEREST

The authors declare no competing interests exist.

## AUTHOR CONTRIBUTIONS

**Deep Jyoti Chapagain:** Conceptualization (equal); Data curation (equal); Formal analysis (equal); Investigation (equal); Methodology (equal); Writing‐original draft (equal); Writing‐review & editing (equal). **Henrik Meilby:** Conceptualization (equal); Formal analysis (equal); Methodology (equal); Project administration (equal); Supervision (equal); Writing‐original draft (equal); Writing‐review & editing (equal). **Chitra Bahadur Baniya:** Supervision (equal); Writing‐review & editing (supporting). **Shanta Budha‐Magar:** Data curation (equal); Writing‐review & editing (supporting). **Suresh Kumar Ghimire:** Conceptualization (equal); Formal analysis (equal); Methodology (equal); Project administration (equal); Supervision (equal); Writing‐original draft (equal); Writing‐review & editing (equal).

## AUTHOR'S CONTRIBUTION

DJC and SKG designed the experiment; DJC and SBM collected the data; DJC, HM, CBB, and SKG analyzed the data and wrote the manuscript.

## Supporting information

Appendix S1

Appendix S2

Data S1

## Data Availability

Data used for the ecological analysis are presented in the paper and the Supplementary material. Data that identify human participants in the study will not be made publicly available. However such data can be made available upon request to the authors.
