# Supplementary material for: Illegal harvesting and livestock grazing threaten the endangered orchid Dactylorhiza hatagirea (D. Don) Soó in Nepalese Himalaya
Source: Ecol Evol. 2021 May 1;11(11):6672–87. doi: 10.1002/ece3.7520 (PMC8207444; doi:10.1002/ece3.7520)
Supplement: Supplementary file 1 — Appendix S1 [file ECE3-11-6672-s002.docx]

**Appendix S1**

Table S1 Summary of environmental variables (topographic and substrate) characterizing three populations each of locally unprotected and locally protected sites of *Dactylorhiza hatagirea* in Api-Nampa Conservation Area (ANCA), Western Nepal and Manang, Central Nepal. Estimates of the topographic and substrate variables are specified as mean ± SE; ranges are also stated for topographic variables.

| **Topographic variables** | **Locally unprotected site (Darchula)** | | | **Locally protected site (Manang)** | | |
| --- | --- | --- | --- | --- | --- | --- |
|  | **Dhauli** | **Nwagidanda** | **Kalidhunga** | **Bhimthang** | **Ponker Hill** | **Salpodanda** |
| Elevation (m a.s.l) | 3604.91±9.09 | 3799.41 ± 3.52 | 3975.62 ± 7.44 | 3712.59 ± 4.67 | 4045.80 ± 10.60 | 4436.50 ± 12.40 |
|  | (3,460-3,750) | (3,750-3,860) | (3,855-4,090) | (3,620-3,790) | (3,880-4,200) | (4,205-4,600) |
| Slope (degree) | 17.20±0.87 | 26.06 ± 1.38 | 10.04 ± 0.58 | 25.50 ± 1.80 | 31.09 ± 1.64 | 36.46 ± 1.58 |
|  | (2-25) | (6-65) | (1-22) | (4-63) | (5-56) | (7-65) |
| Aspect (degree) | 162.30±4.66 | 222±4.56 | 231.83 ± 5.89 | 119.59 ± 5.96 | 166.80 ± 3.78 | 164.67 ± 4.18 |
|  | (90-248) | (132-342) | (20-320) | (30-250) | (100-260) | (104-280) |
| Relative radiation Index (RRI) | 0.92 ± 0.00 | 0.88 ± 0.01 | 0.89 ± 0.00 | 0.81 ± 0.01 | 0.90 ± 0.00 | 0.89 ± 0.00 |
|  | (0.77- 0.99) | (0.38-0.98) | (0.78-0.99) | (0.58-0.99) | (0.55-0.99) | (0.61-0.99) |
| **Substrate variables** |  |  |  |  |  |  |
| Herb cover (%) | 75.10 ± 1.80 | 53.80 ± 3.22 | 61.58 ± 2.70 | 26.32 ± 1.71 | 17.13 ± 1.54 | 46.23 ± 2.40 |
| Grass cover (%) | 2.76 ± 0.36 | 15.82 ± 1.90 | 15.66 ± 2.08 | 1.87 ± 0.14 | 11.84 ± 1.49 | 11.97 ± 0.80 |
| Moss cover (%) | 2.41 ± 0.16 | 6.13 ± 0.52 | 4.58 ± 0.47 | 28.49 ± 1.57 | 27.57 ± 1.86 | 16.10 ± 1.38 |
| Lichen cover (%) | 0.11 ± 0.02 | 1.20 ± 0.17 | 1.16 ± 0.11 | 5.70 ± 0.40 | 9.67 ± 0.63 | 4.10 ± 0.36 |
| Litter cover (%) | 2.92 ± 0.17 | 4.47 ± 0.46 | 2.51 ± 0.16 | 7.66 ± 0.91 | 12.13 ± 0.74 | 8.37 ± 0.74 |
| Rock cover (%) | 4.50 ± 1.09 | 15.10 ± 2.26 | 4.99 ± 0.98 | 20.42 ± 1.98 | 18.11 ± 2.26 | 12.10 ± 1.76 |
| Scree cover (%) | 0.16 ± 0.07 | 0.20 ± 0.11 | 1.41 ± 0.50 | 2.19 ± 0.36 | 1.69 ± 0.34 | 0.22 ± 0.10 |
| Bare ground cover (%) | 12.10 ± 1.30 | 3.37 ± 0.44 | 8.21 ± 1.18 | 7.17 ± 1.44 | 2.02 ± 0.53 | 1.21 ± 0.21 |
