# Supplementary material for: Illegal harvesting and livestock grazing threaten the endangered orchid Dactylorhiza hatagirea (D. Don) Soó in Nepalese Himalaya
Source: Ecol Evol. 2021 May 1;11(11):6672–87. doi: 10.1002/ece3.7520 (PMC8207444; doi:10.1002/ece3.7520)
Supplement: Supplementary file 2 — Appendix S2 [file ECE3-11-6672-s003.docx]

**Appendix S2**

**Set of Models**

**Model 1**

Density _ij_ (of a particular stage of *D. hatagirea*) = a +b (population) + c_1_Harvesting _ij_ + c_2_Herbcover _ij_ + (1|Plot)

**Model 2**

Density _ij_ (of a particular stage of *D. hatagirea*) = a + b (Population) +c_1_Harvesting _ij_ + (1|Plot)

**Model 3**

Density _ij_ (of a particular stage of *D. hatagirea*) = a + b (Population) + c_1_RRI _ij_+ c_2_Harvesting _ij +_ (1|Plot)

**Model 4**

Density _ij_ (of a particular stage of *D. hatagirea*) = a + b (Population) + c_1_Herb cover _ij_+ c_2_ RRI _ij_+ c_3_ Harvesting _ij +_ (1|Plot)

**Model 5**

Density _ij_ (of a particular stage of *D. hatagirea*) = a) + b (Population)+ c_1_Harvesting _ij_ + c_2_ Trampling _ij +_  c_3_ Animal dropping _ij_+ (1|Plot)

**Model 6**

Density _ij_ (of a particular stage of *D. hatagirea*) = a + b(Population)+ c_1_Herb cover _ij_ + c_2_Harvesting _ij_+ c_3_ Trampling _ij+_ c_4_ Animal dropping _ij+_ (1|Plot)

**Model 7**

Density _ij_ (of a particular stage of *D. hatagirea*) = a + b(Population)+ c_1_Herb cover _ij_ + c_2_Harvesting _ij_+ c_3_ Trampling _ij_ + (1|Plot)

**Model 8**

Density _ij_ (of a particular stage of *D. hatagirea*) = a + b (Population) + c_1_ Harvesting _ij_ + c_2_Trampling _ij_ + (1|Plot)

**Model 9**

Density _ij_ (of a particular stage of *D. hatagirea*) = a + b (Population) + c_1_ Animal dropping _ij_ + c_2_ Trampling _ij_ + (1|Plot)

**Model10**

Density _ij_ (of a particular stage of *D. hatagirea*) = a + b (Population) + c_1_Animal dropping _ij_ + (1|Plot)

Where, a (intercept), b (Population) and c_1_…c_3_ are fixed model parameters, i =1…144 is the plot (included as a random effect) and j=1…4 is the sub-plot. Population has six categories (three at each site). Density is expressed as the number of individuals in a given stage category counted within a 1 m^2^ sub-plot.
